# TOR Mediates Stress Responses Through Global Regulation of Metabolome in Plants

**DOI:** 10.3390/ijms26052095

**Published:** 2025-02-27

**Authors:** Lin Yang, Ran Zhang, Huan Zhang, Yingyu Yang, Liwen Fu

**Affiliations:** 1 Shanghai Collaborative Innovation Center of Agri-Seeds, School of Agriculture and Biology, Shanghai Jiao Tong University, Shanghai 200240, China; yanglin2023@sjtu.edu.cn (L.Y.); zr_807@sjtu.edu.cn (R.Z.); zhang_h@sjtu.edu.cn (H.Z.); yangyingyu@sjtu.edu.cn (Y.Y.); 2 Joint Center for Single Cell Biology, School of Agriculture and Biology, Shanghai Jiao Tong University, Shanghai 200240, China

**Keywords:** target of rapamycin (TOR), plant stress responses, biotic stress, abiotic stress, metabolome

## Abstract

The target of rapamycin (TOR) kinase is an evolutionarily conserved atypical Ser/Thr protein kinase present in yeasts, plants, and mammals. In plants, TOR acts as a central signaling hub, playing a pivotal role in the precise orchestration of growth and development. Extensive studies have underscored its significant role in these processes. Recent research has further elucidated TOR’s multifaceted roles in plant stress adaptation. Furthermore, mounting evidence indicates TOR’s role in mediating the plant metabolome. In this review, we will discuss recent findings on the involvement of TOR signaling in plant adaptation to various abiotic and biotic stresses, with a specific focus on TOR-regulated metabolome reprogramming in response to different stresses.

## 1. Introduction

Plants exist in intricate environments where they are continually subjected to a range of abiotic and biotic stresses, such as extreme temperatures, drought, high salinity, and pathogen attacks [1,2]. Given their immobility, plants have evolved a sophisticated signal transduction network to manage these challenging environmental conditions [1,2]. This network facilitates large-scale transcriptional, translational, and metabolic changes, which are crucial for redirecting the plant’s limited energies and resources from growth and development toward defense mechanisms [3]. Consequently, the plants’ stress responses are intricately linked to these adaptive changes, enabling them to survive and thrive despite adverse conditions.

The target of rapamycin (TOR) is a conserved serine/threonine kinase found in eukaryotes and is a member of the phosphoinositide-3 kinase-related protein kinase (PIKK) family [4,5]. Initially identified in the yeast (*Saccharomyces cerevisiae*), TOR was recognized as the target protein of rapamycin, an antibiotic produced by *Streptomyces hygroscopicus* and first discovered in 1975 by Vézina et al. [6]. Subsequent studies have demonstrated the presence of TOR across a variety of non-photosynthetic organisms, including mammals and *Drosophila* [4,5]. Furthermore, TOR has been identified in plants, expanding its known presence across both photosynthetic and non-photosynthetic organisms [4,5].

The *TOR* genes, identified across various species, encode large proteins typically comprising 2400–2500 amino acids, and these proteins exhibit significant similarity among different eukaryotes [4,5]. A typical TOR protein is characterized by several domains arranged sequentially from the *N*-terminus to the C-terminus: the HEAT (Huntingtin, elongation factor 3, subunit A of PP2A, TOR1) tandem repeat sequence, FAT (FRAP/ATM/TTRAP) domain, FRB (FKBP-rapamycin binding) domain, protein kinase domain, and FATC (Carboxyl-terminal FAT domain) domain [4,5]. The HEAT repeat sequences are crucial for facilitating protein-protein interactions and membrane binding [4,5]. Both the FAT and FATC domains play roles in protein interactions and kinase activation [4,5]. The FRB domain serves as the target for rapamycin, a specific inhibitor of TOR kinase. Rapamycin binds to the FKBP12 protein (FK506-binding protein 12 kDa), forming a complex that specifically attaches to the FRB domain, thereby inhibiting TOR activity [4,5].

TOR operates primarily through two complexes, TORC1 and TORC2, which are distinguished by their composition and function [4,7,8]. TORC1 is composed of TOR, Lethal with Sec Thirteen 8 (LST8 in plants and yeast, mLST8 in mammals), and the Regulatory-Associated Protein of TOR (RAPTOR in mammals and plants, KOG1 in yeast); in contrast, TORC2 includes TOR, LST8/mLST8, Raptor Independent Companion of TOR (RICTOR), and Stress-activated MAP Kinase Interacting Protein 1 (SIN1) [4,9,10,11,12]. In yeast, there are two TOR genes, *TOR1* and *TOR2*, with TOR1 or TOR2 capable of forming a TORC1 complex, whereas only TOR2 can form a TORC2 complex [13]. In mammals, a single *TOR* gene is involved in both TORC1 and TORC2 complexes [14]. In plants, only the typical TORC1 complex has been identified. However, TORC2 may not be conserved. It is possible that other proteins with similar functions could substitute for RICTOR and SIN1, suggesting the potential existence of another form of TORC2 complex in plants [13]. Notably, the FKBP12-rapamycin complex binds to the conserved FRB domain in TOR kinase, inhibiting TORC1 activity, but it cannot bind to TORC2 complexes due to the spatial occupation by mSIN1 or AVO1 [4].

Over the past two and a half decades since its discovery, extensive research in both yeast and mammalian model systems has revealed TOR’s fundamental role as a central signaling hub [8,15,16,17]. By integrating multiple major signaling pathways, TOR coordinates responses to nutrient availability, cellular energy status, growth factors, and various stressors [8,15,16,17]. Through this comprehensive integration of signals, TOR effectively orchestrates both cellular and organismal physiology, underscoring its essential function in maintaining homeostasis and regulating diverse biological processes [8,15,16,17].

In *Arabidopsis*, null *tor* mutants exhibit an embryonic lethal phenotype, and TOR in *Arabidopsis* has traditionally been regarded as resistant to rapamycin, which has hindered progress in plant TOR research. Some groundbreaking work addressed these challenges by developing a system to detect TOR activity in plants and creating the estradiol-inducible *tor* mutant, *tor-es* [18,19]. This innovation facilitated the discovery that plant TOR is indeed sensitive to rapamycin, albeit requiring higher concentrations [18,19]. Subsequently, the introduction of new-generation ATP-competitive chemical inhibitors targeting TOR kinases, such as AZD8055, Torin1, Torin2, and KU0063794, has significantly advanced plant TOR research [20]. TOR has been extensively reported to mediate various aspects of plant growth and development by regulating translation, transcription, autophagy, and primary and secondary metabolism. Additionally, numerous studies have highlighted TOR’s crucial role in plant responses to both biotic and abiotic stresses [21].

In this review, we will explore recent advances in TOR-mediated stress responses. Specifically, as the significance of TOR-regulated primary and secondary metabolism becomes increasingly recognized, we will examine the specific role of TOR-modulated metabolic reprogramming in plant responses to diverse biotic and abiotic stresses.

## 2. Insight on TOR-Mediated Stress Responses via Metabolome Reprogramming

### 2.1. TOR Is Widely Involved in Plants Responses to Various Environmental Stresses

**Figure 1:**
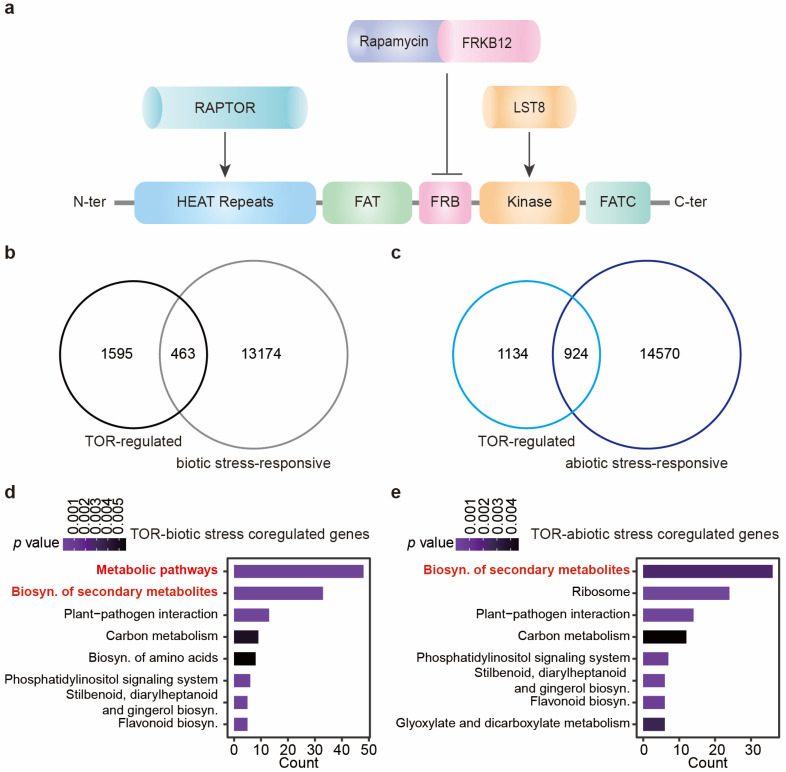
TOR regulates a large number of stress-responsive genes. (**a**) Structure and complex of TOR kinase in plants. (**b**) Overlapping genes between TOR-regulated and biotic stress-responsive genes. (**c**) Overlapping genes between TOR-regulated and abiotic stress-responsive genes. (**d**) KEGG analysis of overlapping genes in (**b**). (**e**) KEGG analysis of overlapping genes in (**c**).

As illustrated in Figure 1a, a typical TOR protein in plants is sequentially arranged from the *N*-terminus to the C-terminus as follows: HEAT repeats, FAT domain, FRB domain, kinase domain, and FATC domain [4,5]. The amino acid sequences of TOR kinases across different plant species exhibit high levels of identity. For example, *Arabidopsis* TOR (Q9FR53) and tomato TOR (A0A3Q7ERV7), both dicotyledonous plants, share an identity of 78.9% and a similarity of 86.9%. In comparison, *At*TOR (*Arabidopsis* TOR) and *Os*TOR (rice TOR, Q0DJS1), with rice being a monocotyledonous plant, show an identity of 72.9% and a similarity of 82.2%. The high degree of identity and similarity in TOR amino acid sequences among various different monocotyledonous and dicotyledonous plants underscores the strong conservation of its function. To date, only one TOR complex has been identified in plants, consisting of Raptor, LST8, and TOR itself (Figure 1a, [4,5]).

The function of TOR has long been associated with regulating protein translation [22]. However, recent discoveries have revealed that TOR can either directly phosphorylate specific transcription factors, such as E2Fa and E2Fb, or indirectly regulate the stability of others, including EIN3, in ethylene signaling [19,23]. These findings underscore TOR’s crucial role in gene transcription regulation. Furthermore, transcriptome analysis indicates that TOR facilitates significant transcriptomic reprogramming [19,23].

To elucidate the role of TOR in regulating plant responses to stress, we conducted a comparative analysis of genes regulated by TOR (identified through treatment with a TOR inhibitor, Torin2) [24] and those affected by various biotic and abiotic stresses in tomatoes. Specifically, we pooled genes differentially expressed in response to various kinds of biotic stresses into one set, including *Pseudomonas syringae* pv. tomato DC3000-, *Phytophthora parasitica*-, and herbivore-regulated genes (Table S1) [25,26,27]; similarly, genes differentially expressed in response to drought, salt, cold, and heat were consolidated to form the abiotic stress-responsive gene set (Table S2) [28,29,30,31]. Upon analysis of gene expression patterns, we found substantial overlap between TOR-regulated genes and stress-responsive gene sets. Specifically, approximately one-fifth (463/2058) of the TOR-mediated genes showed concordance with biotic stress-responsive genes, while approximately half (924/2058) overlapped with genes regulated under abiotic stress conditions (Figure 1b,c). This significant overlap suggests a broad involvement of TOR in modulating plant stress responses.

To elucidate the biological pathways implicated by these intersecting genes, we conducted a KEGG (Kyoto Encyclopedia of Genes and Genomes) pathway enrichment analysis using the two overlapping gene sets. Notably, when examining the intersection between TOR-regulated genes and those responsive to biotic or abiotic stresses, the “metabolic pathways” and “biosynthesis of secondary metabolites” emerged as the most significantly enriched pathways (Figure 1d,e). Transcriptional changes are relatively direct processes that occur within hours, typically preceding metabolic changes. It is possible that TOR may affect plant stress response through transcriptome reprogramming, followed by metabolome reprogramming, ultimately leading to physiological adaptations to stress conditions.

Based on the conclusions drawn from the transcriptome analysis, we will first summarize recent findings concerning TOR-regulated plant responses to abiotic and biotic stresses. Subsequently, we will specifically discuss the role of TOR-mediated metabolic changes in plant stress adaptation.

### 2.2. TOR-Regulated Plant Responses to Abiotic Stress

Throughout their life cycle, plants face a range of abiotic stresses, including cold, heat, drought, and salt [1]. To survive and thrive under these fluctuating environmental conditions, plants must swiftly respond to diverse stress signals [1]. A pivotal component of this adaptive response is the TOR signaling pathway, which is instrumental in regulating plant reactions to these various abiotic stresses [4].

Abnormal temperatures have a profound impact on biological processes in plants, influencing growth, development, metabolism, protein translation, and gene expression [32,33]. Recent studies indicate that TOR plays a crucial role in mediating the effects of both low and high temperatures.

In *Arabidopsis*, exposure to low temperatures initially causes a rapid reduction in TOR activity within the first hour, followed by a recovery after a longer duration [34,35,36]. This fluctuation pattern in TOR activity is also observed in tomatoes, suggesting that it represents a conserved mechanism across different species [24]. Furthermore, cold stress not only affects TOR activity at the post-translational level but also influences its expression levels. Specifically, cold exposure induces TOR expression between 1- and 3-h post-treatment, whereas prolonged exposure results in the repression of its expression [34]. These findings collectively indicate that cold stress modulates TOR both at the post-translational and transcriptional levels.

TOR plays a complex role in regulating cold responses in *Arabidopsis*. Research by Wang et al. indicates that TOR negatively regulates cold responses using 18-day-old seedlings, whereas findings by Dong et al. suggest that TOR promotes these responses using 7-week-old plants, with Thyroid Adenoma Associated (THADA) acting upstream of TOR in this process [36,37]. The findings indicate that TOR might regulate cold responses in a growth stage-dependent manner in plants. Additionally, the FERONIA-ROP2-TOR module has been shown to enhance root hair growth at low temperatures, underscoring TOR’s multifaceted role in plant cold resistance [35]. In tomatoes, low temperatures rapidly and transiently suppress TOR activity, which subsequently activates the transcriptional activity of its direct substrate, PGH1 [24]. This activation leads to increased expression of the *C-REPEAT-BINDING FACTOR 1* (*CBF1*) gene, a key player in cold acclimation and stress response [24]. This, in turn, activates the expression of cold stress response-related genes and regulates metabolic reprogramming, enhancing the accumulation of cold-resistant metabolites and thereby improving tomato cold resistance [24]. These findings provide comprehensive insights into the intricate regulatory mechanisms of TOR in plant cold stress response.

Elevated temperatures also adversely affect grain crop yield and quality. In perennial ryegrass (*Lolium perenne*), *TOR* expression is upregulated under high-temperature stress [36]. In *Arabidopsis*, glucose via TOR governs the transcriptome reprogramming of a large number of genes involved in heat stress protection [38,39,40]. The E2Fa (E2 PROMOTER BINDING FACTOR a) transcription factor, activated by the glucose-TOR module, is shown to activate root apical meristem and promote root growth [19]. Meanwhile, recent studies also indicate that the glucose-TOR-activated E2Fa also binds to the promoters of HSF genes and enhances the expression of heat-responsive genes [38,39,40]. Specifically, the Glc-TOR-E2Fa module activates the expression of *HIKESHI-LIKE PROTEIN1* (*HLP1*), which binds to the promoters of glucose-regulated HS-responsive genes [38,39,40]. Furthermore, Glc-TOR-E2Fa activates the expression of *ARABIDOPSIS TRITHORAX 1* (*ATX1*), encoding an H3K4 methyltransferase already shown to regulate H3K4me3 levels at the promoters of HS recovery genes. Also, glucose through TOR promotes the recruitment of histone H3 acetylation marks at the promoters of HS genes to induce their transcription, leading to thermotolerance. This Glc-TOR-mediated histone acetylation is facilitated through HAC1 [38,39,40].

Salt and osmotic stress induce physiological and biochemical changes, leading to metabolic irregularities and growth cessation [41,42]. High NaCl concentration increases TOR activity, while mannitol-induced drought stress inhibits TOR activity [34]. Constitutive overexpression of TOR enhances salt and osmotic stress tolerance in *Arabidopsis thaliana*, showing improved performance in root growth, fresh weight, and lateral root density [43,44]. YAK1 in *Arabidopsis* acts as a positive regulator of ABA-mediated drought response. Under favorable growth conditions, TOR inhibits YAK1 activity, thereby negatively regulating ABA signaling transduction and ABA-mediated drought response [45,46]. Furthermore, oxidative stress is reported to enhance TOR activity during short-time treatment while inhibiting TOR activity [34].

Abscisic acid (ABA) is a crucial stress phytohormone that plays a significant role in plant responses to various abiotic stresses, including cold, salt, osmotic, and drought conditions [47]. A sophisticated reciprocal regulation exists between ABA and TOR signaling, which balances stress adaptation and plant growth [48,49]. Under stress conditions, ABA-activated SnRK2 kinases directly interact with and phosphorylate RAPTOR, a core component of the TOR complex (TORC1) [48,49]. This interaction leads to the dissociation of TORC1, inhibition of TOR kinase activity, and subsequent repression of plant growth [48,49]. Conversely, under favorable conditions, activated TOR kinase phosphorylates PYLs, preventing ABA from binding to these receptors [48,49]. This inactivation of SnRK2 promotes plant growth [48,49]. Salicylic acid (SA) is another essential stress phytohormone involved in various abiotic stress responses. A recent study conducted on tomatoes demonstrated that inhibiting TOR under cold stress conditions significantly increased the levels of SA and its derivatives [24].

Autophagy serves as a fundamental degradation and recycling pathway for cytoplasmic substances in eukaryotes and is induced by various stressors, with a significant connection to the TOR signaling pathway [50]. In plants, inhibition of TOR signaling can initiate autophagy, while TOR overexpression can prevent it under nutrient deficiencies, salt, and drought stress, though not under oxidative or endoplasmic reticulum stress [51,52,53,54]. SNF1-related kinase 1 (SnRK1) plays a crucial role in regulating autophagy upstream of TOR during nutrient deficiency, osmotic pressure, and salt stress. In contrast, the regulation of autophagy by oxidative and endoplasmic reticulum stress is dependent on SnRK1 rather than TOR [52,53,54]. Although the role of plant TOR as a regulator of autophagy in response to nutrients and abiotic stress conditions is established [51,52,53,54,55], the precise connection between TOR and the core components in autophagy remains unclear. Notably, recent interactome and phosphoproteomics analyses have identified several autophagy-related proteins as potential substrates of TOR kinase, suggesting a more intricate involvement of TOR in autophagy regulation [21,56].

### 2.3. TOR-Regulated Plant Responses to Biotic Stress

In addition to various abiotic stresses, plants are also threatened by a range of biotic stresses, including viruses, bacteria, fungi, nematodes, and insects. To counter these biotic threats, plants have developed a sophisticated immune system comprising two layers of defense: pattern-triggered immunity (PTI) and effector-triggered immunity (ETI) [57,58]. PTI is activated by molecular patterns associated with microbes, pathogens, nematodes, herbivores, and parasites, known as MAMPs, PAMPs, NAMPs, HAMPs, and ParAMPs, respectively. In contrast, pathogen- and parasite-secreted proteins, termed effectors, are perceived by host-derived immunogenic molecules to trigger ETI [57,58]. This dual-layered immune system enables plants to effectively recognize and respond to a wide array of biotic stressors. In this review, plant defense responses against pathogens are a major focus of discussion.

The mechanisms by which plant TOR responds to various pathogens remain largely unexplored. However, recent studies have begun to elucidate potential pathways of regulation. For instance, it has been demonstrated that biotic stresses may influence TOR at the transcriptional level. Specifically, a study on tomatoes revealed that the transcription factor *Sl*MYC2 directly binds to the promoter region of *SlTOR*, thereby activating its transcription [59]. Given that jasmonic acid (JA) is a crucial phytohormone involved in biotic stress responses and that MYC2 is the central transcription factor in JA signaling, it is plausible that certain biotic stresses increase JA levels, which in turn activate MYC2 and subsequently enhance *TOR* transcription [59,60]. In contrast, at the translational level, the *Pseudomonas* effector AvrRpm1 has been found to suppress *TOR* expression [61]. Furthermore, the type III effector AWR5, when expressed in yeast, exhibits a function similar to that of rapamycin, suggesting a potential role in TOR regulation [62]. These findings indicate a complex interplay between biotic stressors and TOR signaling, warranting further investigation.

In *Solanaceae* plants such as tomato and tobacco, the inhibition of TOR activity, either through the use of the TOR inhibitor Torin2 or via Virus-Induced Gene Silencing (VIGS), has been shown to enhance plant defense against various pathogens. In tomatoes, specifically, the repression of TOR activity strengthens defense mechanisms against *Botrytis cinerea*, *Alternaria alternata,* and *Xanthomonas euvesicatoria* [63]. Similarly, in tobacco, silencing *TOR* increases resistance to *Xanthomonas euvesicatoria* and the tobacco mosaic virus [63]. The study indicates that TOR-regulated plant immunity is dependent on SA [63]. In tomatoes, TOR coordinates cytokinin (CK) and gibberellin (GA) signaling, which mediates both development and defense [64]. The effectiveness of disease resistance mediated by TOR inhibition varies with the developmental stage, being absent in highly morphogenetic leaves but most potent in mature, differentiated ones [64]. Marash’s research demonstrated that inoculating tomatoes with pathogens at different CK/GA ratios revealed that higher CK/GA levels suppressed TOR activity and enhanced resistance, whereas lower CK/GA levels had the opposite effect [64]. This suggests that the differential regulation of TOR could modulate the development-defense trade-off in plants. In rice, TOR negatively regulates both SA- and JA-dependent immune responses and pathogen-triggered immunity (PTI) [65]. SA and JA are known to have antagonistic effects on plant immunity; SA positively regulates immunity against biotrophic and hemibiotrophic pathogens, while JA is effective against necrotrophic pathogens [66]. This study provides valuable insights into how TOR may mitigate the competing effects of these two plant hormones, thereby regulating plant responses to a diverse array of pathogens. Furthermore, the research in rice established a sophisticated model illustrating that TOR balances plant growth and defense, promoting growth while repressing defense responses. In *Arabidopsis*, TOR inhibition via a TOR inhibitor increased resistance to *Fusarium graminearum* [67], and the suppression of TOR expression enhanced resistance to *Pseudomonas syringae* and oomycete pathogens [61]. Additionally, viruses can hijack TOR signaling to facilitate their replication [68], and *TOR* silencing or inhibition has been shown to promote resistance against the watermelon mosaic virus [69]. Intriguingly, TOR kinases in both dicotyledonous and monocotyledonous plants exhibit a conserved function in suppressing defense responses, highlighting the evolutionary preservation of this regulatory mechanism across diverse plant species.

### 2.4. TOR-Modulated Metabolic Reprogramming Contributes to Plant Stress Responses

Mammalian mTOR-mediated metabolic reprogramming is extensively studied, and its dysregulation is implicated in severe human diseases like cancer, type 2 diabetes/obesity, and neurodegenerative disorders [70].

Similarly, in plants, multiple independent studies have demonstrated that TOR tightly regulates the plant metabolome [46,71,72,73,74,75,76,77,78,79,80,81,82,83,84,85,86,87]. Of particular interest is the relationship between TOR-regulated metabolites and plant stress responses, as it has been clearly shown that stress triggers numerous metabolic changes in plants [88]. To systematically investigate this connection, we analyzed the metabolomic data from a comprehensive study by Song et al. in *Arabidopsis* [83]. Their analysis of an inducible tor mutant (*tor-es*) identified 141 differentially accumulated metabolites (DAMs). Through an extensive literature review of these DAMs’ biological functions, we found that 25 metabolites were associated with abiotic stress responses, 11 with biotic stress responses, and 3 metabolites (3-Methylmalic acid, amentoflavone, and caffeine) were involved in both response pathways, highlighting the significant role of TOR in plant stress metabolism. The functions of the 33 DAMs involved in plant stress responses are summarized in Table 1. Based on the table, TOR appears to regulate plant stress responses by controlling the synthesis and accumulation of stress-response metabolites.

More direct evidence for TOR-mediated metabolic reprogramming in stress resistance comes from a study in tomato. In tomatoes, cold stress inhibits TOR kinase activity, which triggers a cascade of molecular responses that enhance cold resistance through metabolic reprogramming [24]. This inhibition prevents TOR kinase from phosphorylating the transcription factor PGH1, and the resulting dephosphorylated PGH1 exhibits increased transcriptional activity, leading to enhanced expression of the core cold stress response gene *CBF1* [24]. Through this TOR-PGH1-CBF1 axis, extensive transcriptional reprogramming occurs, particularly affecting genes encoding key metabolic enzymes [24]. Specifically, while genes involved in amino acid synthesis are downregulated, those crucial for protective compound synthesis are upregulated, including *CS2* and *ICS1* in the shikimate synthesis pathway and *OTC*, *ADC1*, and *ADC2* in the putrescine synthesis pathway [24]. Metabolomic analyses confirm that TOR inhibition under cold stress conditions substantially alters the metabolic landscape [24]. These alterations manifest as reduced amino acid synthesis coupled with enhanced production of cryoprotective compounds, including salicylic acid and its derivatives, flavonoids, and putrescine [24]. This metabolic shift suggests that TOR functions as a central regulator in cold stress response by redistributing carbon skeletons from primary metabolism toward the synthesis of cryoprotective compounds, thereby enabling plants to adapt to cold conditions [24].

## 3. Discussion

Plants face continuous challenges from diverse environmental stresses, which has led to the evolution of sophisticated signaling networks for survival and adaptation. The TOR has emerged as a central regulatory component within these networks, playing crucial roles in stress response mechanisms. Extensive research from multiple laboratories has demonstrated TOR’s widespread involvement in mediating plants’ responses to various environmental stresses. Despite this substantial body of work, there remains a significant gap in our understanding of how TOR-regulated metabolic alterations connect to TOR-mediated stress responses. Therefore, investigating TOR-orchestrated stress responses from a metabolic perspective offers promising new insights into deciphering these complex regulatory mechanisms.

TOR has been demonstrated to influence the accumulation of diverse metabolites in plants, though the underlying mechanisms remain largely unexplored. TOR may exert both direct and indirect control over metabolite biosynthesis. In terms of indirect regulation, TOR can trigger comprehensive transcriptome reprogramming, leading to the modulation of stress-resistant compound accumulation [24]. This often occurs at the expense of growth- and development-related metabolites, reflecting a resource allocation strategy through which plants maintain a delicate balance between defense and growth under limited energy resources [24]. Additionally, TOR can directly regulate metabolic pathways by phosphorylating key enzymatic components. This direct regulatory mechanism is exemplified in a recent tomato study, where TOR was found to phosphorylate PGH1, a crucial glycolytic enzyme that also functions as a transcription factor in *CBF1* expression regulation [24].

Under natural conditions, plants are exposed to multiple concurrent environmental stresses rather than experiencing individual stressors in isolation. The TOR signaling pathway demonstrates a remarkable capacity to orchestrate multiple stress responses through its regulation of diverse compounds, as comprehensively illustrated in Table 1. These compounds are integral components of various stress response mechanisms, positioning TOR as an ideal molecular framework for investigating the complex interplay of multiple environmental stresses and their combined effects on plant physiology.

**Figure 2:**
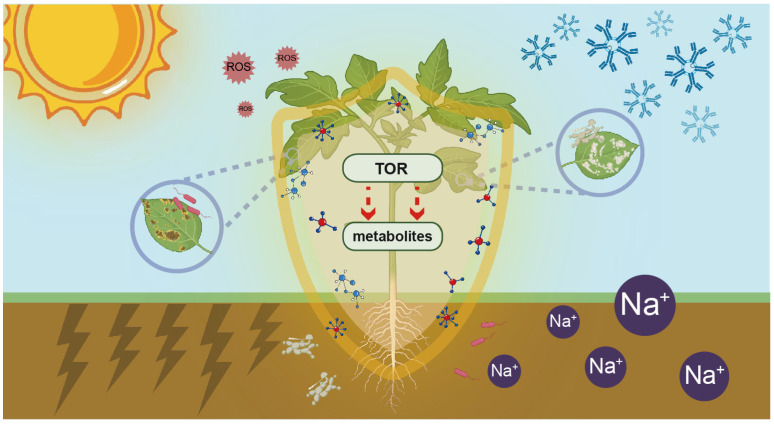
TOR-mediated metabolic reprogramming enhances plant resilience against diverse abiotic and biotic stresses. Plants, as sessile organisms, are continuously exposed to a wide spectrum of environmental challenges. These challenges encompass both abiotic stresses, including salinity, drought, oxidative conditions, and extreme temperatures, and biotic stresses from various organisms such as microbes, pathogens, nematodes, herbivores, and parasites. Within the complex plant signaling network, TOR functions as a central regulatory hub that orchestrates metabolic reprogramming to enhance stress resilience. This master regulator integrates diverse upstream stress signals and modulates plant metabolism through multiple mechanisms. Specifically, TOR regulates transcriptional responses to enhance the production of stress-resistant metabolites, directly modifies the activity of key metabolic enzymes across various pathways, and employs additional regulatory mechanisms. Consequently, the accumulated stress-resistant compounds establish a protective biochemical “shield”, thereby significantly enhancing plant resilience against multiple environmental stressors. The sun symbolizes heat stress, while the snowflakes represent cold stress. Cracks in the ground signify drought stress, and the presence of Na^+^ reflects salt stress. ROS denotes oxidative stress, and microbes on the leaves or the roots indicate the invasion of plant pathogens.

The mechanism by which TOR regulates stress responses through metabolic reprogramming is illustrated in Figure 2.

Although the connection between TOR-mediated stress adaptation and metabolome reprogramming has been established, the precise mechanisms underlying this relationship remain elusive. Current evidence suggests that transcriptome reprogramming drives large-scale metabolome reprogramming, which ultimately influences stress adaptation indirectly. However, more direct TOR substrates responsible for initiating these metabolic changes have yet to be identified. Future research should focus on screening for more catalytic enzymes that act as TOR substrates, employing advanced techniques such as phosphoproteomics or interactome analysis. These efforts will undoubtedly provide critical insights into the specific mechanisms by which TOR regulates stress adaptation through metabolome reprogramming, advancing our understanding of this complex regulatory network.

## Figures and Tables

**Table 1:** TOR-mediated stress-related DAMs in *Arabidopsis*. The data originated from Song et al. [83], and the functions of the 33 stress-related DAMs were determined through comprehensive literature analysis.

Compounds	Class	Up- or Down-Regulated in *tor-es*	Function	References
1,5-Diaminopentane	Phenolamides	up	Drought, oxidative stress, heavy metal stress	[89,90]
2-Aminoadipic acid (L-Homoglutamic acid)	Amino acids and derivatives	up	Oxidative stress, drought, salt	[91,92,93,94]
3-Indoleacetonitrile	Indole derivatives	down	Pathogen attack	[95,96,97]
3-Methylmalic acid	Organic acids and derivatives	up	Salt, drought, biotic stress	[98,99,100]
4-Hydroxycoumarin	Phenylpropanoids	down	Pathogen attack	[101]
5-Aminolevulinate	Organic acids and derivatives	up	Drought, salt, heavy metal	[102,103,104,105,106,107,108]
5-Oxoproline	Amino acids and derivatives	up	Heat stress	[109,110]
6-Aminocaproic acid	Organic acids and derivatives	up	Salt stress	[111]
Amentoflavone	Flavone	up	Temperature, light, drought, biotic stress	[112]
Caffeic acid	Phenylpropanoids	up	Salt stress	[113,114]
Caffeine	Alkaloids	down	Drought, biotic stress	[115,116]
Camalexin	Alkaloids	up	Pathogen attack	[117]
Chlorogenic acid (3-O-Caffeoylquinic acid)	Organic acids and derivatives	down	Cold stress	[118]
Chrysoeriol	Flavone	up	Oxidative stress	[119,120]
Citramalate	Organic acids and derivatives	up	Pathogen attack	[121]
Citric acid monohydrate	Organic acids and derivatives	up	Heavy metal	[122,123,124,125,126]
Citric acid	Organic acids and derivatives	up	Heavy metal	[122,123,124,125,126]
DIMBOA glucoside	Others	up	Biotic stress	[127,128]
Genistein 7-O-Glucoside (Genistin)	Isoflavone	up	Drought	[129]
Homogentisic acid	Organic acids and derivatives	up	Abiotic stress, ABA signaling	[130]
L-(-)-Tyrosine	Amino acids and derivatives	up	Abiotic stress	[131]
L-Ascorbate	Vitamins and derivatives	up	Abiotic stress	[132]
L-Homoserine	Amino acids and derivatives	up	Drought, salt stress	[133,134]
L-Pipecolic acid	Amino acids and derivatives	up	Biotic stress	[135]
*N*-Acetyl-L-phenylalanine	Amino acids and derivatives	up	Cold, drought	[136,137]
Narirutin	Flavone	up	Heavy metal stress, oxidative stress	[138,139]
*N*-p-Coumaroyl agmatine	Phenolamides	up	Biotic stress	[140]
Quinic acid	Organic acids and derivatives	up	Abiotic stress	[141]
S-Allyl-L-cysteine	Amino acids and derivatives	up	Heavy metal stress	[142,143]
Sinapyl alcohol	Phenylpropanoids	up	Flooding stress, cold stress	[144,145]
Tricin	Flavone	down	Cold, drought, salt stress	[146,147]
Umbelliferone	Phenylpropanoids	down	Pathogen attack	[148,149]
Xanthohumol	Flavanone	up	Drought stress	[150]

## Data Availability

The original contributions presented in this study are included in the article/Supplementary Material. Further inquiries can be directed to the corresponding author.

## Supplementary Materials

The following supporting information can be downloaded at https://www.mdpi.com/article/10.3390/ijms26052095/s1.
